# Supplementary material for: Unraveling Fe(II)-Oxidizing Mechanisms in a Facultative Fe(II) Oxidizer, Sideroxydans lithotrophicus Strain ES-1, via Culturing, Transcriptomics, and Reverse Transcription-Quantitative PCR
Source: Appl Environ Microbiol. 2022 Jan 25;88(2):e01595-21. doi: 10.1128/AEM.01595-21 (PMC8788666; doi:10.1128/AEM.01595-21)
Supplement: Supplemental file 1 — Fig. S1 to S5, Tables S1 to S7. Download AEM.01595-21-s0001.pdf, PDF file, 0.9 MB [file aem.01595-21-s0001.pdf]

Supplemental materials

Unraveling Fe(II)-oxidizing mechanisms in a facultative Fe(II) oxidizer *Sideroxydans lithotrophicus* ES-1 via culturing, transcriptomics, and RT-qPCR

Nanqing Zhou,[a] Jessica L. Keffer,[b] Shawn W. Polson,[c,d] Clara S. Chan[a,b*]

School of Marine Science and Policy, University of Delaware, Newark, DE, USA[a]
Department of Earth Sciences, University of Delaware, Newark, DE, USA[b]
Department of Computer and Information Sciences, Newark, DE, USA[c]
Center for Bioinformatics and Computational Biology, Newark, DE, USA[d]

*Corresponding author:

Clara S. Chan: cschan@udel.edu

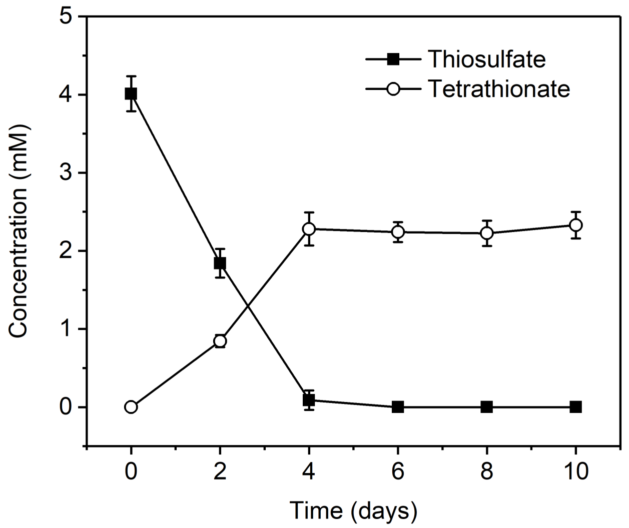

Figure S1. Thiosulfate oxidation by ES-1 produces tetrathionate (in a 1:2 ratio).

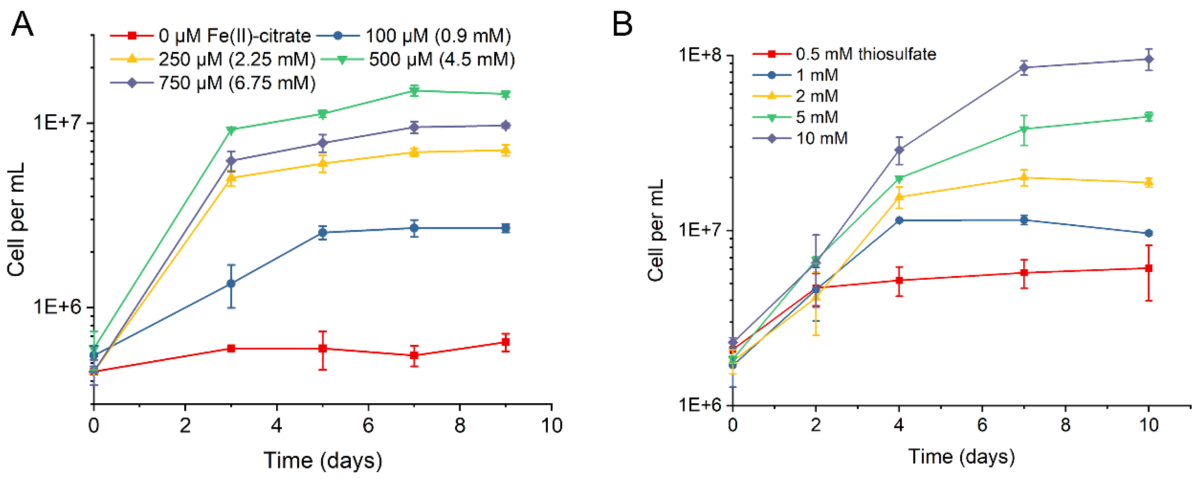

Figure S2. (A) Cell growth at different concentrations of Fe(II)-citrate (given as a daily supplement); (B) Cell growth at different concentrations of thiosulfate (one-time supplement). The numbers in the parentheses of the legend in (A) represent total Fe(II) addition during the 9-day culturing. The 0 µM Fe(II)-citrate represents citrate only. The total amount of substrate added in 100 µM (navy blue), 250 µM (yellow) and 500 µM (green) groups in Fe(II)-citrate culture is comparable to 1 mM (navy blue), 2 mM (yellow) and 5 mM (green) groups in thiosulfate. Thus, these groups can be used to compare the ES-1 biomass yield using different substrates.

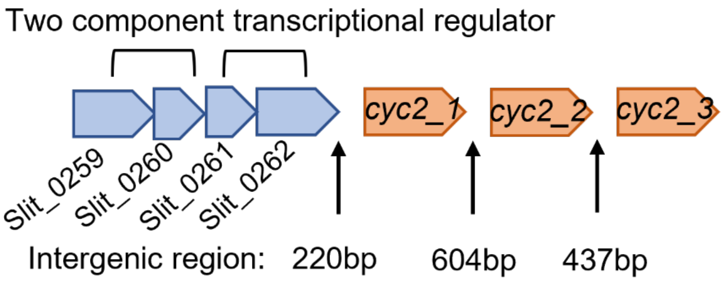

Figure S3. *cyc2* gene map

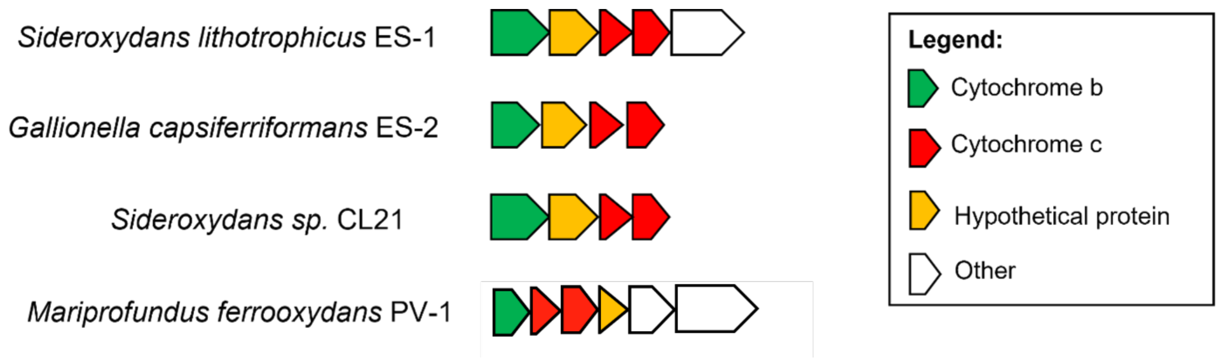

Figure S4. Conserved cytochrome-containing gene cluster in different FeOB isolates. Locus tag of the genes: ES-1 (Slit_1321-1325), ES-2 (Galf_0405-0408), CL21 (SIDCL21_2123-2126) and PV-1 (SPV1_01612-01617).

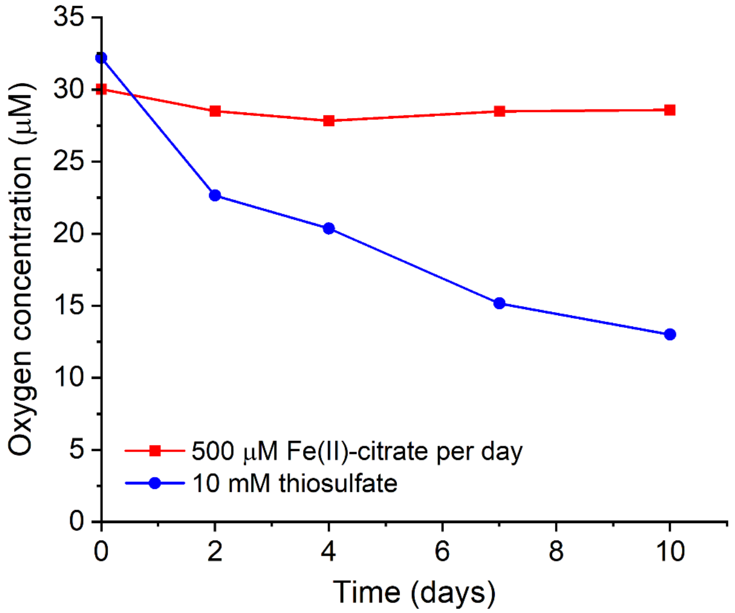

Figure S5. Oxygen concentration change in Fe(II)-citrate and thiosulfate cultures. Oxygen concentration was measured every 48 hours immediately before flushing the headspace with 2% O_2 gas mix. Data points are the average of two replicates.

Table S1. Expression of potential sulfur oxidases

Gene	Growth Condition	Average Norm. TPM	Norm. TPM Range	percentile	Lowest	Highest
soxXYZ AB (Slit_1700-1696)	Fe-midlog	1.0	0.5-1.4	61.7-86.1	*soxB*	*soxA*
	Fe-latelog	1.1	0.6-1.5	62.2-87.6	*soxB*	*soxA*
	Fe-stationary	0.3	0.2-0.5	31.4-58.0	*soxY*	*soxA*
	S-midlog	6.3	3.9-7.7	94.8-98.1	*soxB*	*soxX*
	S-latelog	9.7	5.1-12.1	95.8-98.8	*soxB*	*soxX*
	S-stationary	10.2	5.0-13.1	96.6-98.9	*soxB*	*soxX*
	15min	2.2	1.6-2.8	86.0-91.5	*soxY*	*soxX*
	35min	1.8	1.4-2.3	84.5-90.1	*soxY*	*soxA*
	90min	1.4	0.9-1.9	74.2-87.9	*soxY*	*soxX*
dsrABE FHCMK LJOPN (Slit_1686-1673)	Fe-miglog	1.2	0.1-3.1	20.9-94.1	*dsrN*	*dsrA*
	Fe-latelog	0.9	0.2-2.2	21.3-91.8	*dsrN*	*dsrC*
	Fe-stationary	0.7	0.2-1.8	26.1-84.2	*dsrN*	*dsrC*
	S-midlog	2.9	0.9-6.5	68.5-97.1	*dsrN*	*dsrC*
	S-latelog	7.1	1.1-22.5	74.0-99.2	*dsrN*	*dsrC*
	S-staionary	2.7	0.6-6.7	57.2-97.6	*dsrN*	*dsrC*
	15min	1.1	0.4-3.2	58.5-92.6	*dsrE*	*dsrB*
	35min	2.5	0.5-8.8	61.1-95.9	*dsrN*	*dsrH*
	90min	2.2	0.4-4.9	52.2-94.8	*dsrN*	*dsrA*
tsdAB (Slit_1877-1878)	Fe-miglog	0.4	0.4-0.5	57.1-57.6	*tsdA*	*tsdB*
	Fe-latelog	0.2	0.2-0.2	27.7-31.4	*tsdB*	*tsdA*
	Fe-stationary	0.5	0.5-0.6	50.1-61.1	*tsdB*	*tsdA*
	S-midlog	0.1	0.1-0.1	8.7-14.0	*tsdB*	*tsdA*
	S-latelog	0.4	0.3-0.4	35.9-38.1	*tsdA*	*tsdB*
	S-staionary	0.1	0.1-0.1	14.1-15.7	*tsdB*	*tsdA*
	15min	0.1	0-0.2	8.4-25.8	*tsdB*	*tsdA*
	35min	0.2	0.1-0.2	24.6-36.4	*tsdB*	*tsdA*
	90min	0.5	0.4-0.6	47.6-61.5	*tsdB*	*tsdA*

Table S2. Relative expression level of *cyc2* genes and *mtoA* to *gyrB* using RT-qPCR

Growth condition	*cyc2_1*(Slit_0263)		*cyc2_2*(Slit_0264)		*cyc2_3*(Slit_0265)		*mtoA*	
	Mean	SD	Mean	SD	Mean	SD	Mean	SD
Fe-midlog	21.51	5.81	18.45	4.83	5.48	1.68	0.15	0.01
Fe-latelog	45.28	13.88	13.79	1.13	2.64	0.39	0.09	0.01
Fe-stationary	33.67	26.69	8.75	1.27	3.51	2.04	0.09	0.01
S-midlog	86.76	2.50	1.91	0.12	0.42	0.05	0.13	0.01
S-latelog	101.39	23.75	5.18	0.91	0.89	0.05	0.13	0.12
S-stationary	106.31	41.46	3.57	1.84	0.72	0.09	0.19	0.11
15 min	145.23	38.49	2.47	0.30	1.31	0.48	0.74	0.23
35 min	57.35	16.90	2.98	0.44	1.46	0.67	0.07	0.03
90 min	100.16	19.75	1.85	0.29	0.57	0.16	0.85	0.71

Mean: The mean relative expression value calculated from three biological replicates.
SD: standard deviation of the relative expression values of three biological replicates.

Table S3. Expression and differential expression analysis of *cyc2* and *mto* by transcriptome

Gene	Growth condition	Norm.TPM	Rank	Percentile	log_2FC
cyc2_1* *(Slit_0263)	Fe-midlog	17.6	15	99.5	-1.18
	Fe-latelog	40.6	4	99.9	ns
	Fe-stationary	22.2	20	99.3	-1.11
	S-midlog	57.8	5	99.8	
	S-latelog	50.7	6	99.8	
	S-stationary	51.0	6	99.8	
	15min	61.0	2	99.9	0.65
	35min	61.5	3	100.0	0.77
	90min	68.6	2	99.9	0.76
cyc2_2* *(Slit_0264)	Fe-midlog	16.0	18	99.4	4.40
	Fe-latelog	12.4	27	99.1	2.72
	Fe-stationary	8.3	72	97.6	2.24
	S-midlog	1.1	758	74.6	
	S-latelog	2.3	319	89.3	
	S-stationary	2.0	351	88.3	
	15min	1.3	490	83.6	ns
	35min	2.7	261	91.3	1.01
	90min	1.4	511	82.9	ns
cyc2_3* *(Slit_0265)	Fe-midlog	6.1	94	96.9	4.65
	Fe-latelog	2.9	180	94.0	2.70
	Fe-stationary	3.4	201	93.3	3.18
	S-midlog	0.4	1971	33.9	
	S-latelog	0.6	1364	54.3	
	S-stationary	0.4	1645	44.8	
	15min	0.8	760	74.5	1.31
	35min	1.6	421	85.9	2.41
	90min	0.5	1262	57.7	0.50
mtoA* *(Slit_2497)	Fe-midlog	0.0	2721	8.7	2.98
	Fe-latelog	0.0	2826	5.2	-3.69
	Fe-stationary	0.0	2879	3.4	ns
	S-midlog	0.0	2932	1.6	
	S-latelog	0.4	1863	37.5	
	S-stationary	0.0	2893	3.0	
	15min	0.1	2250	24.5	3.20
	35min	0.0	2719	8.8	ns
	90min	0.5	1301	56.4	4.89
mtoB* *(Slit_2496)	Fe-midlog	0.1	2706	9.2	-2.88
	Fe-latelog	0.0	2749	7.6	-0.93
	Fe-stationary	0.0	2807	5.8	
	S-midlog	0.1	2740	8.1	
	S-latelog	0.4	1743	41.5	
	S-stationary	0.1	2744	7.9	
	15min	0.1	2350	21.1	1.55
	35min	0.5	1094	63.3	3.64
	90min	0.8	788	73.6	4.15

cymA (Slit_2495)	Fe-midlog	0.0	2754	7.6	ns
	Fe-latelog	0.0	2765	7.2	-2.44
	Fe-stationary	0.0	2827	5.1	ns
	S-midlog	0.1	2760	7.4	
	S-latelog	0.3	2199	26.2	
	S-stationary	0.0	2830	5.0	
	15min	0.0	2812	5.6	
	35min	0.7	839	71.8	5.05
	90min	2.3	310	89.6	6.54
mtoD (Slit_2498)	Fe-midlog	0.0	2756	7.5	ns
	Fe-latelog	0.0	2818	5.4	N/A
	Fe-stationary	0.0	2844	4.6	ns
	S-midlog	0.0	2870	3.7	
	S-latelog	0.4	1853	37.8	
	S-stationary	0.0	2907	2.4	
	15min	0.1	2523	15.3	2.93
	35min	0.0	2932	1.6	N/A
	90min	1.1	618	79.3	6.42

Note: log_2FC represents log_2fold change in Fe(II)-citrate vs. thiosulfate culture in long-term; different time points (15 min, 35 min and 90 min) after adding $FeCl_2$ vs time 0 in short-term. ns stands for not significant, which means the p value from DESeq analysis is greater than 0.05. N/A means there is 0 count in the biological replicates.

Table S4. Gene differential expression analysis of other cytochromes

**Group**	**Gene**	**Heme binding sites**	**Percentile in Fe(II)-citrate culture**			**Percentile in thiosulfate culture**			**Percentile after adding $FeCl_2$**			**Log2FC (Fe(II)-citrate vs. thiosulfate)**			**Log2FC (after vs. before adding $FeCl_2$)**		
			midlog	latelog	stationary	midlog	latelog	stationary	15min	35min	90min	midlog	latelog	stationary	15min	35min	90min
PCC3 cluster1	Slit_0867	0	2.9	4.3	4.3	11.3	9.3	10.5	6.8	5.4	5.6	-2.74	-1.91	-2.13	-1.35	-1.68	-1.76
	Slit_0868	17	6.9	6.3	6.5	11.6	13.2	12.4	6.7	9.2	6.5	-1.31	-1.85	-1.76	-1.72	-1.00	-1.87
	Slit_0869	21	12.4	12.3	9.5	24.5	24.2	26.9	15.3	18.8	13.9	-1.20	-1.17	-1.92	-1.26	-0.94	-1.47
	Slit_0870	0	7.3	8.2	7.5	16.1	17.2	14.5	12.1	10.3	16.6	-1.68	-1.58	-1.54	ns	ns	ns
PCC3 cluster2	Slit_1446	0	72.8	77.0	83.4	86.6	85.9	86.5	83.4	82.1	84.3	-0.82	-0.77	ns	ns	ns	ns
	Slit_1447	24	65.8	64.5	68.7	77.7	76.4	75.0	62.3	59.9	65.4	-0.41	-0.70	ns	-0.65	-0.82	NS
	Slit_1448	28	62.0	58.7	49.1	73.9	74.3	73.8	47.2	38.0	40.9	-0.51	-0.78	-1.11	-1.22	-1.71	-1.51
	Slit_1449	0	46.0	46.0	45.6	61.5	62.8	62.3	45.1	36.8	35.4	-0.69	-0.64	-0.74	-0.79	-1.25	-1.20
Other cytoch-romes	Slit_1321	0	85.0	80.3	89.6	67.2	74.2	74.3	93.1	91.9	88.8	1.16	ns	1.26	2.26	2.11	1.46
	Slit_1322	0	96.1	94.9	94.8	79.6	83.1	85.1	93.8	94.0	91.5	2.43	1.43	1.48	1.71	2.00	1.18
	Slit_1323	1	95.1	92.6	91.7	70.0	82.4	74.8	88.5	92.9	90.7	2.60	1.03	1.53	1.38	2.34	1.69
	Slit_1324	2	97.3	96.3	95.6	87.3	91.4	92.2	92.4	95.5	94.8	2.34	0.93	1.10	0.65	1.91	1.19
	Slit_1353	1	99.7	99.7	99.8	98.8	99.4	99.3	97.8	97.0	96.4	1.38	ns	0.70	ns	ns	-0.99
	Slit_2042	1	92.4	99.0	97.0	97.2	97.3	95.2	98.7	98.0	98.3	-0.78	ns	ns	3.06	2.83	2.76
	Slit_2657	2	98.8	99.2	98.3	99.2	99.1	99.2	98.6	98.9	98.9	-0.28	-0.06	-0.87	0.70	1.09	0.71

Note: The genes with 0 heme binding sites encode for porins or other types of cytochromes (not c-type). Slit_0867 and Slit_1449 encode for porins. Slit_0870 and Slit_1446 are potential inner membrane proteins. Slit_1321 encodes for a cytochrome b_{561} while Slit_1322 is a hypothetical protein. “ns” stands for not significant, which means the p value from DESeq analysis is greater than 0.05.

Table S5. Expression and differential expression analysis of *ccoN* and *ccoO*

Gene	Growth condition	Norm. TPM	Percentile	Log2FC
ccoN_ proximal	Fe-midlog	8.5	98.2	1.20
	Fe-latelog	5.9	97.8	ns
	Fe-stationary	4.5	95.3	-0.56
	S-midlog	5.4	96.8	
	S-latelog	7.6	97.6	
	S-stationary	7.5	97.9	
	15 min	5.2	95.0	ns
	35 min	3.9	93.4	ns
	90 min	1.0	77.7	-2.56
ccoO_ proximal	Fe-midlog	8.0	97.8	1.20
	Fe-latelog	6.5	98.0	ns
	Fe-stationary	4.5	95.2	ns
	S-midlog	5.1	96.5	
	S-latelog	8.0	97.9	
	S-stationary	7.2	97.8	
	15 min	3.4	93.1	ns
	35 min	7.6	95.6	ns
	90 min	1.4	83.7	-2.04
ccoN_ distal	Fe-midlog	14.5	99.3	-0.20
	Fe-latelog	17.7	99.4	ns
	Fe-stationary	8.7	97.8	ns
	S-midlog	24.3	99.5	
	S-latelog	22.1	99.3	
	S-stationary	23.5	99.4	
	15 min	21.2	98.3	ns
	35 min	17.1	94.6	ns
	90 min	19.5	98.0	ns
ccoO_ distal	Fe-midlog	13.0	99.2	-0.30
	Fe-latelog	15.3	99.2	ns
	Fe-stationary	10.5	98.4	-0.93
	S-midlog	23.3	99.4	
	S-latelog	21.6	99.3	
	S-stationary	21.7	99.3	
	15 min	25.3	98.8	0.62
	35 min	15.3	97.3	ns
	90 min	21.0	98.4	ns

Note: log_2FC represents log_2fold change in Fe(II)-citrate vs. thiosulfate culture in long-term; different time points (15 min, 35 min and 90 min) after adding $FeCl_2$ vs time 0 in short-term. ns stands for not significant, which means the p value from DESeq analysis is greater than 0.05.

Table S6. Expression and differential expression analysis of putative Complex III genes

Gene	Growth condition	Average norm. TPM	Norm. TPM range	Max percentile	Lowest	Highest	Max log_2FC
bc1 complex (Slit_0130-0132)	Fe-midlog	3.6	3.4-3.8	94.9	*Slit_0132*	*Slit_0130*	0.94
	Fe-latelog	2.8	2.6-2.9	93.8	*Slit_0131*	*Slit_0130*	-0.13
	Fe-stationary	2.7	2.1-3.0	92.7	*Slit_0131*	*Slit_0130*	0.06
	S-midlog	3.3	2.8-3.8	94.8	*Slit_0131*	*Slit_0130*	
	S-latelog	4.2	3.5-5.3	96.0	*Slit_0131*	*Slit_0130*	
	S-stationary	3.4	2.6-4.3	95.9	*Slit_0131*	*Slit_0130*	
	15min	3.9	3.2-4.6	94.5	*Slit_0132*	*Slit_0130*	1.00
	35min	4.5	3.0-6.6	95.2	*Slit_0131*	*Slit_0132*	1.59
	90min	3.1	2.0-4.1	94.0	*Slit_0131*	*Slit_0130*	0.39
ACIII (Slit_0640-0646)	Fe-midlog	1.8	0.9-2.5	92.7	*Slit_0641*	*Slit_0645*	2.13
	Fe-latelog	1.9	1.4-2.6	93.0	*Slit_0641*	*Slit_0642*	2.00
	Fe-stationary	2.0	1.1-2.9	92.4	*Slit_0641*	*Slit_0642*	2.36
	S-midlog	0.8	0.6-1.0	72.0	*Slit_0644*	*Slit_0645*	
	S-latelog	0.9	0.5-1.2	78.2	*Slit_0644*	*Slit_0645*	
	S-stationary	0.5	0.4-0.7	64.2	*Slit_0644*	*Slit_0645*	
	15min	16.6	4.3-34.1	99.4	*Slit_0646*	*Slit_0640*	6.41
	35min	20.9	14.1-43.0	99.6	*Slit_0643*	*Slit_0640*	6.84
	90min	17.2	11.9-27.7	99.2	*Slit_0643*	*Slit_0645*	5.64

Note: Average norm. TPM is calculated from averaging the constitutive normalized TPM values of the genes in the gene cluster. Max log_2FC represents the maximum log2fold change in the gene cluster. log_2fold change in Fe(II)-citrate vs. thiosulfate culture in long-term; different time points (15 min, 35 min and 90 min) after adding $FeCl_2$ vs time 0 in short-term.

Table S7. RT-qPCR primers for target genes

Gene	Forward primer	Reverse primer	T_a (ºC)	Length (bp)
Slit_0263	GCGACGTTACCGCATTGTTAGC	GCAACATTTGCGTTGTACAGGTT	55	160
Slit_0264	ATGCCGGTCGGCTTCTATGCT	ACCACCGTTGGGAGCCAT	59	93
Slit_0265	CAGACATTCGTTGACGTGCAG	TGCACCGCATACTGGCGC	55	93
mtoA	CCACCTGAAGGGTAATGTGGAT	GTGCGATTGGTACCCTTGTGG	55	135
gyrB	CACTACGAGATCTTGGCTAAGC	GCTCTTGTTCATGTACTCGAC	55	150

Note: T_a represents the annealing temperature.
